# Evaluating knowledge and attitudes towards dengue fever and its vaccination in China: A questionnaire-based study in healthcare practitioners

**DOI:** 10.1371/journal.pntd.0013530

**Published:** 2026-08-05

**Authors:** IpMan Lam, Ye Yao, Yuxiang Sun, Yilan Xia, Yihan Lu

**Affiliations:** 1 School of Public Health, Yale University, New Haven, Connecticut, United States of America; 2 Department of Biostatistics, School of Public Health, Fudan University, Shanghai, China; 3 Shanghai Institute of Infectious Diseases and Biosecurity, Fudan University, Shanghai, China; 4 Department of Epidemiology, Ministry of Education Key Laboratory of Public Health Safety (Fudan University), School of Public Health, Fudan University, Shanghai, China; LSTM: Liverpool School of Tropical Medicine, UNITED KINGDOM OF GREAT BRITAIN AND NORTHERN IRELAND

## Abstract

Dengue, a mosquito-borne tropical disease caused by the dengue virus, has seen a significant increase in global incidence in recent decades. China has experienced a rapid rise in dengue cases since 2013, posing a serious threat to population health and increasing economic burden. This study aimed to evaluate the knowledge of, and attitudes toward, dengue fever and dengue vaccination among healthcare practitioners in China. A cross-sectional study was conducted using an online questionnaire survey targeting medical professionals in Zhejiang, Yunnan, and Hainan provinces, which are endemic areas for dengue virus infection in China. The questionnaire assessed participants’ understanding of dengue virus transmission, high-risk groups, symptoms, and knowledge of and attitudes toward dengue vaccines. Results showed that 98.6% of participants reported having prior knowledge of dengue virus. Knowledge of at-risk populations was strongly associated with actual dengue knowledge and vaccine attitudes, which may drive vaccine acceptance. Most respondents (71.0%) held neutral attitudes toward dengue vaccination, followed by 20.8% who held positive attitudes and 8.2% who held negative attitudes. Individuals with neutral attitudes were the most critical in determining vaccine acceptance. Among those who accepted vaccination, 56.1% held neutral attitudes, and among those who refused vaccination, 84.3% held neutral attitudes. Whilst most respondents (87.6%) reported that they would vaccinate susceptible populations against dengue, only 7.5% were aware that approved dengue vaccines exist. This study provides valuable insights into current knowledge of, and attitudes toward, dengue fever and vaccination among healthcare practitioners in China. Associations between specific knowledge domains, vaccine attitudes, and vaccine acceptance highlight directions for educational campaigns and vaccine promotion.

## Author summary

Evaluating knowledge of, and attitudes toward, dengue fever and dengue vaccination among healthcare practitioners in China is crucial for informing future vaccination policy development. Because China has not introduced any dengue vaccines, endemic regions rely heavily on non-pharmaceutical interventions and post-infection treatment. This article highlights the misconceptions and concerns of dengue and dengue vaccines within the medical community in China, which are potential targets for interventions and communication strategies by public health authorities to ultimately reduce the health burden. This study also identifies commonly shared misinformation, providing a useful reference for endemic regions to better understand dengue virus and the benefits and health requirements of dengue vaccination before making vaccination decisions. We believe that expanded research on, and eventual introduction of, dengue vaccines would benefit the Chinese population. The findings of this study offered empirical insights for decision-making and designing effective dengue-related education campaigns that can maximize their impact on vaccine attitudes and acceptance.

## Introduction

Dengue virus, a member of the *Flaviviridae* family and the genus *Orthoflavivirus*, is the etiological agent that causes dengue fever. Dengue fever is a mosquito-borne disease that exerts its greatest disease burden in tropical and subtropical regions, yet it now has an increasingly significant impact on global health. The virus is primarily transmitted through the bites of infected female mosquitoes, predominantly of the species *Aedes aegypti* and *Aedes albopictus*, to a susceptible human [1]. The geographical distribution of these mosquitoes reflects the regions where dengue virus is endemic, including various parts of the subtropics and tropics, with notable prevalence in urban and semi-urban areas [1].

Dengue virus infection has a wide spectrum of clinical presentations, ranging from asymptomatic or mild illnesses to severe flu-like symptoms, and is characterized by high fever, headache, pain behind the eyes, muscle and joint pains, and skin rashes [2]. Some infection cases can also progress to severe dengue, formerly known as dengue hemorrhagic fever (DHF) or dengue shock syndrome (DSS). These severe forms may be life-threatening and are characterized by plasma leakage, fluid accumulation, respiratory distress, severe bleeding, or organ impairment [2].

The global incidence of dengue has increased dramatically in recent decades. The global number of dengue cases reached 14.1 million in 2024 – a figure that is double the previous historic high recorded in 2023 [3]. The reasons for this rise are multifaceted, including increased urbanization, population growth, increased global tourism after the COVID-19 pandemic, insufficient public health measures to raise awareness and improve water sanitation, and climate change, which has expanded the habitable environment for the mosquito vectors. The lack of a specific and effective antiviral treatment for dengue virus infection underscores the importance of preventive measures and supportive care. Primary prevention focuses on vector control measures to reduce the mosquito population and minimize human-vector contact. In addition, intensive research continues into the development and deployment of dengue vaccines, despite persistent challenges concerning vaccine efficacy and safety, most notably antibody-dependent enhancement (ADE) [4].

Understanding the biology of the dengue virus, the pathogenesis of the infection, and the complexities of the immune response to the virus is critical to developing effective treatments and preventive strategies. As the global burden of dengue continues to grow, it is imperative for the international community to strengthen surveillance, improve vector control, invest in vaccine research, and ensure that healthcare systems are prepared to manage both the expected and the more severe presentations of the infection.

Since the outbreak of dengue fever in Guangdong in the 1970s, dengue fever epidemics have occurred every year in China, and the number of cases has increased rapidly after 2013 [5]. From 2016 to 2018, the number of reported cases of dengue fever in China increased from 2050 to 5136, more than doubling [6,^7].^ In 2019, before the emergence of SARS-CoV-2, the number of dengue fever infections reached a historical peak of 22,188 cases [8].

In 2019, the number of reported dengue fever cases in China increased significantly, posing a serious threat to population health and increasing economic burden. Dengue fever cases resulted in a total of 46,805,064 yuan in direct health spending in 2019 [9]. By comparison, the cost of prevention and control measures was 6,934,378 yuan, including simple and feasible measures such as removing wastewater from potted plants and cleaning streets [9]. As the pandemic gradually mitigated, there was an expected resurgence of dengue cases in China and worldwide that paralleled the increased interprovincial travel and expanding mosquito habitats [10].

In order to effectively prevent and control the spread of dengue fever, vaccination is an important and effective tool. Currently, two quadrivalent dengue vaccines have been approved for marketing in some countries, namely Dengvaxia by Sanofi Pasteur and Qdenga by Takeda Pharmaceuticals. Both vaccines offer some protection against the four serotypes of dengue virus but differ in terms of effectiveness, safety, and suitable populations. The effectiveness of Dengvaxia is mainly reflected in the protection of patients who have recovered from dengue fever. Its overall efficacy is 82%, with 79% efficacy against the endpoint of hospitalization and 84% efficacy in patients with severe dengue fever [11]. However, Dengvaxia may increase the risk of severe dengue fever in people who have never been infected with dengue fever, hence is only recommended for people from 9 to 16 years old and with a history of dengue fever infection by the Centers for Disease Control and Prevention (CDC) [12].

Qdenga provided data to support its effectiveness and safety and is recommended for a wider range of people with varied infection histories. Qdenga can be administered to anyone aged over 4 years old, regardless of previous dengue infection history, with an overall efficacy of 80.2% against all dengue serotypes over 12 months, 90.4% efficacy against endpoint of hospitalization, and 85.9% efficacy against dengue hemorrhagic fever patients [13]. Qdenga is effective against dengue serotypes 1 and 2, with an efficacy of 78% against hospitalized patients with dengue serotype 1 and 100% against hospitalized patients with dengue serotype 2 [13,14]. Dengvaxia can provide antibody protection for at least 6 years, while Qdenga can provide immune protection for at least 4.5 years [15,16]. Hence, both vaccines require regular booster shots in epidemic regions.

At present, China has not introduced these vaccine products for use in Chinese population. This study aims to evaluate the knowledge and attitudes towards dengue fever and dengue vaccination among healthcare practitioners in China. This study aimed to offer empirical insights into healthcare workers’ knowledge and attitudes regarding dengue fever, which may inform future research on dengue vaccine introduction and help tailor prevention strategies to local needs in China.

## Methods

### Ethical considerations

This study was approved by the Institutional Review Board (IRB) of the Fudan University School of Public Health (IRB 00002408 and FWA 00002399) under IRB #2023-10-1085. All respondents accessed the online questionnaire and read the informed consent. They clicked “agree” to the informed consent, which demonstrated they provided informed consent, and then filled out the questionnaire.

### Study design and participants

This cross-sectional study employed a questionnaire survey to assess knowledge of, and attitudes toward, dengue fever and its vaccination among healthcare practitioners in China. Data were collected in December 2023. The target population included medical professionals working in Zhejiang, Yunnan, and Hainan provinces, which are endemic areas for dengue virus infection. Participants were recruited passively through publicly accessible online platforms distributed across various healthcare settings, including Chinese Centers for Disease Control and Prevention (China CDC), hospitals (both infectious and non-infectious disease units), and community-level healthcare facilities (e.g., preventive health care departments).

### Questionnaire development and administration

The questionnaire was developed by the research team to assess participants’ basic information, understanding of dengue virus and vaccines, and attitudes towards dengue vaccination. The questionnaire consisted of three main sections:

(1) Demographics: This section collected data on participants’ nature of work unit, age, education level, specific work department or line, and years of working experience.
(2) Understanding of dengue virus and vaccines: This section assessed participants’ knowledge of dengue virus transmission routes, epidemic-prone areas in China, high-risk groups for dengue fever and severe dengue, common symptoms of infection, and understanding of dengue vaccines, including global approval status, applicable groups, safety, effectiveness, key characteristics, and antibody-dependent enhancement (ADE). This section divided participants into two groups according to their responses: “knowledgeable” and “non-knowledgeable”. Participants who answered “Yes” to the question “Do you know dengue?” were categorized as “knowledgeable”, whereas who answered “No” were categorized as “non-knowledgeable”.
(3) Attitudes towards dengue vaccination: This section investigated participants’ anticipated recommendations for dengue vaccination among different groups, awareness of the use of dengue vaccine, and acceptance of the vaccine.

The questionnaire was administered online through the Questionnaire Star platform. The online questionnaire was disseminated to institutions and hospitals, allowing participants to access and complete the survey electronically.

### Data analysis

Categorical variables were presented as frequencies and percentages, while continuous variables were presented as means and standard deviations. Chi-square tests were used to compare categorical variables, and t-tests were employed for continuous variables in the baseline characteristics assessment.

Actual vaccine knowledge was defined as correctly answering at least 4 out of the 6 questions on basic dengue vaccine knowledge and reporting knowledge of antibody-dependent enhancement. A positive vaccine attitude was defined as expressing positivity toward 8 out of the 12 vaccine statements, and a negative vaccine attitude was defined as expressing negativity toward 8 out of the 12 vaccine statements; the remaining participants were categorized as neutral. The overall dengue-related knowledge was stratified into six domains: (1) Knowledge of dengue transmission, (2) Knowledge of mosquito vectors, (3) Knowledge of high at-risk population for dengue (exposure), (4) Knowledge of high at-risk population for severe dengue (severity), (5) Knowledge of infection symptoms, and (6) Knowledge of possible infection complications. To evaluate the associations between perceived and actual knowledge of dengue, as well as the relationships among perceived knowledge, actual knowledge, and vaccine attitudes, odds ratios (ORs) and 95% confidence intervals (CIs) were calculated using univariate logistic regression models and maximum likelihood estimate (MLE). Chi-square tests were utilized for independent observations and expected cell counts greater than 5. Independent t-tests were performed with confirmed independence of observations and homogeneity of variances, and the study sample size (N = 1047) was sufficiently large that the tests were robust to mild violations of normality. Due to a small number of zero cells (< 0.6% of the data), a Haldane-Anscombe correction (adding 0.5) was applied for variables with cell counts of 0 to enable odds ratio calculation; results with very wide confidence intervals were interpreted with caution.

All statistical analyses were performed using SAS software (version 9.4). A two-tailed p-value of less than 0.05 was considered statistically significant. Figures were constructed using R Studio and GraphPad Prism 10.

## Results

A total of 1,047 healthcare practitioners were enrolled in the study. A substantial majority of participants (n = 1,032; 98.6%) reported prior knowledge of dengue virus, while 15 participants (1.4%) indicated a lack of such knowledge. Years of professional experience averaged 3.1 (SD = 1.5) in the knowledgeable group and 3.7 (SD = 1.4) in the group without dengue knowledge (p = 0.119) (S1 Table).

Dengue knowledge did not vary significantly by occupational settings (p = 0.066). Participants who reported dengue knowledge and those who did not were largely distributed between the China CDC and community health service centers. Educational attainment differed significantly between the two groups (p = 0.012). Most participants in both groups had completed undergraduate education, including 74.3% of the dengue-knowledgeable group and 66.7% of those without dengue knowledge (S1 Table). In addition, 5.5% (n = 57) of participants in the knowledgeable group and 6.7% (n = 1) of participants in the non-dengue-knowledgeable group held a postgraduate degree (S1 Table).

There were no statistically significant differences between the two groups across the assessed baseline variables except for educational level, suggesting a relatively balanced distribution of participants with respect to dengue knowledge.

1. Perceived knowledge and actual knowledge on dengue virus infection

To further assess healthcare practitioners’ actual dengue knowledge, we compared the odds of correct responses between those who perceived themselves as knowledgeable and those who did not. For factors with statistically significant odds ratios, participants who perceived themselves as well-versed in dengue were more likely to have accurate knowledge about the disease than those who did not consider themselves knowledgeable (OR = 1.380).

Stratification of dengue-related knowledge into specific domains revealed differences in understanding between the two knowledge groups (Table 1). The mean OR obtained for each domain was 1.169 for transmission knowledge, 1.567 for mosquito vector knowledge, 1.110 for high at-risk populations for exposure to infection, 1.328 for high at-risk populations for severe dengue, 1.358 for infection symptoms, and 1.878 for potential complications. Overall, participants who reported perceived knowledge were more likely to answer dengue knowledge items correctly. However, misconceptions or misunderstandings were observed for specific items. For example, participants were unable to correctly recognize that sexual transmission is not a route of dengue virus transmission (OR = 0.269), and that jaundice is not a common symptom of dengue infection (OR = 0.297). It should be noted that most of the observed positive and negative associations between perceived knowledge and actual dengue knowledge were not statistically significant (Table 1).

**Table 1:** Likelihood of actual dengue knowledge among participants with perceived dengue knowledge.

Variables	Says to know dengue, n (%)		Says to not know dengue, n (%)		Odds Ratio ^a^ (95% CI)
	Actually knows	Actually doesn’t know	Actually knows	Actually doesn’t know	
Knowledge of dengue transmission					
Sexual transmission^a^	922 (89.3)	110 (10.7)	15 (100.0)	0 (0.0)	0.269 (0.02, 4.53)
Mosquito bite^a^	1024 (99.2)	8 (0.8)	15 (100.0)	0 (0.0)	3.888 (0.21, 70.38)
Direct contact	839 (81.3)	193 (18.7)	12 (80.0)	3 (20.0)	1.087 (0.30, 3.89)
Indirect contact	875 (84.8)	157 (15.2)	14 (93.3)	1 (6.7)	0.398 (0.05, 3.05)
Saliva transmission	891 (86.3)	141 (13.7)	14 (93.3)	1 (6.7)	0.451 (0.06, 3.46)
Blood transmission	629 (61.0)	403 (39.1)	10 (66.7)	5 (33.3)	0.780 (0.26, 2.30)
Vertical transmission	104 (10.1)	928 (89.9)	1 (6.7)	14 (93.3)	1.569 (0.20, 2.68)
Human-mosquito	322 (31.2)	710 (68.8)	5 (33.3)	10 (66.7)	0.907 (0.31, 2.68)
Knowledge of mosquito vectors					
*Anopheles sinensis*	822 (80.2)	203 (19.8)	12 (80.0)	3 (20.0)	1.012 (0.28, 3.62)
*Anopheles anthropophila*	952 (92.9)	73 (7.1)	13 (86.7)	2 (13.3)	2.006 (0.44, 9.06)
*Anopheles anopheles*	960 (93.7)	65 (6.3)	13 (86.7)	2 (13.3)	2.272 (0.50, 10.28)
*Culex pipiens*	932 (90.9)	93 (9.1)	14 (93.3)	1 (6.7)	0.716 (0.09, 5.50)
*Culex tritaenorhynchus* ^a^	896 (87.4)	129 (12.6)	15 (100.0)	0 (0.0)	0.223 (0.01, 3.75)
*Aedes albopictus*	916 (88.8)	109 (10.6)	12 (80.0)	3 (20.0)	2.101 (0.58, 7.56)
*Aedes aegypti*	819 (79.9)	206 (20.1)	9 (60.0)	6 (40.0)	2.650 (0.93, 7.53)
*Chironomid*	980 (95.6)	45 (4.4)	14 (93.3)	1 (6.7)	1.556 (0.20, 12.09)
Knowledge of high at-risk population for dengue (exposure)					
Elderly	406 (39.3)	626 (60.7)	8 (53.3)	7 (46.7)	0.567 (0.20, 1.58)
Infants and young children	425 (41.2)	607 (58.8)	8 (53.3)	7 (46.7)	0.613 (0.22, 1.70)
Pregnant women	570 (55.2)	462 (44.8)	11 (73.3)	4 (26.7)	0.449 (0.14, 1.42)
Patients with underlying diseases	434 (42.1)	598 (58.0)	8 (53.3)	7 (46.7)	0.635 (0.23, 1.76)
Persons with immune deficiency	359 (34.8)	673 (65.2)	4 (26.7)	11 (73.3)	1.467 (0.46, 4.64)
Re-infected persons with new dengue virus serotype	559 (54.2)	473 (45.8)	9 (60.0)	6 (40.0)	0.788 (0.28, 2.23)
Smoker^a^	872 (84.5)	160 (15.5)	15 (100.0)	0 (0.0)	0.175 (0.01, 2.95)
Persons with malnutrition	678 (65.7)	354 (34.3)	12 (80.0)	3 (20.0)	0.476 (0.13, 1.71)
Outdoor workers	749 (72.6)	283 (27.4)	8 (53.3)	7 (46.7)	2.316 (0.83, 6.45)
Returning travelers	564 (54.7)	468 (45.4)	6 (40.0)	9 (60.0)	1.808 (0.64, 5.12)
Knowledge of high at-risk population for severe dengue (severity)					
Elderly	806 (78.1)	226 (21.9)	9 (60.0)	6 (40.0)	2.378 (0.84, 6.75)
Infants and young children	693 (67.2)	339 (32.9)	7 (46.7)	8 (53.3)	2.336 (0.84, 6.50)
Pregnant women	560 (54.3)	472 (45.7)	4 (26.7)	11 (73.3)	3.263 (1.03, 10.31)
Patients with underlying diseases	835 (80.9)	197 (19.1)	13 (86.7)	2 (13.3)	0.652 (0.15, 2.91)
Persons with immune deficiency	845 (81.9)	187 (18.1)	13 (86.7)	2 (13.3)	0.695 (0.16, 3.11)
Re-infected persons with new dengue virus serotype	522 (50.6)	510 (49.4)	7 (46.7)	8 (53.3)	1.170 (0.42, 3.25)
Smoker^a^	901 (87.3)	131 (12.7)	15 (100.0)	0 (0.0)	0.221 (0.01, 3.72)
Persons with malnutrition	420 (40.7)	612 (59.3)	4 (26.7)	11 (73.3)	1.887 (0.60, 5.97)
Outdoor workers	739 (71.6)	293 (28.4)	13 (86.7)	2 (13.3)	0.388 (0.09, 1.73)
Returning travelers	825 (79.9)	207 (20.1)	14 (93.3)	1 (6.7)	0.285 (0.04, 2.18)
Knowledge of infection symptoms					
Fever^a^	1018 (98.6)	14 (1.4)	15 (100.0)	0 (0.0)	2.266 (0.13, 39.70)
Nausea/Vomiting	547 (53.0)	485 (47.0)	6 (40.0)	9 (60.0)	1.692 (0.60, 4.79)
Pain behind eyes	706 (68.4)	326 (31.6)	9 (60.0)	6 (40.0)	1.444 (0.51, 4.09)
Runny nose	754 (73.1)	278 (26.9)	11 (73.3)	4 (26.7)	0.986 (0.31, 3.12)
Abdominal pain	433 (42.0)	599 (58.0)	3 (20.0)	12 (80.0)	2.891 (0.81, 10.31)
Jaundice	832 (80.6)	200 (19.4)	14 (93.3)	1 (6.7)	0.297 (0.04, 2.27)
Rash	765 (74.1)	267 (25.9)	12 (80.0)	3 (20.0)	0.716 (0.20, 2.56)
Bleeding gums	481 (46.6)	551 (53.4)	7 (46.7)	8 (53.3)	0.998 (0.36, 2.77)
Bone and joint pain	743 (72.0)	289 (28.0)	11 (73.3)	4 (26.7)	0.935 (0.30, 2.96)
Knowledge of possible infection complications					
Acute myocarditis/ Acute heart failure	939 (91.0)	93 (9.0)	13 (86.7)	2 (13.3)	1.553 (0.35, 6.99)
Pulmonary failure	511 (49.5)	521 (50.5)	5 (33.3)	10 (66.7)	1.962 (0.67, 5.78)
Liver failure	715 (69.3)	317 (30.7)	10 (66.7)	5 (33.3)	1.128 (0.38, 3.33)
Acute renal failure	802 (77.7)	230 (22.3)	10 (66.7)	5 (33.3)	1.743 (0.59, 5.15)
Encephalopathy/ Encephalitis	664 (64.3)	368 (35.7)	7 (46.7)	8 (53.3)	2.062 (0.74, 5.73)
Acute gastroenteritis	312 (30.2)	720 (69.8)	2 (13.3)	13 (86.7)	2.817 (0.63, 12.56)

^a^Haldane-Ascombre correction is applied by adding 0.5 to variables with cells containing 0 counts for odds ratio calculation. Wide 95% confidence intervals reflect the small subgroup sizes.

Within the domain of dengue transmission knowledge, respondents with perceived knowledge were more likely to correctly identify mosquito bites (OR = 3.888) and vertical transmission (mother-to-fetus) (OR = 1.569) as possible routes of dengue transmission and to understand that direct contact is not a possible mechanism (OR = 1.087). Within the domain of mosquito vectors, respondents with perceived knowledge were more likely to correctly identify *Aedes aegypti* (OR = 2.650) and *Aedes albopictus* (OR = 2.101). Respondents with perceived knowledge were also more likely to identify common symptoms of dengue infection, including fever (OR = 2.266), nausea/vomiting (OR = 1.692), pain behind the eyes (OR = 1.444), and abdominal pain (OR = 2.891). Respondents with perceived knowledge were consistently more likely to have a correct understanding across all parameters within the domain of potential complications (1.10 < OR < 2.90). Of all the knowledge items assessed, only one variable was statistically significant: participants who reported perceived knowledge of dengue had significantly higher odds of knowing that pregnant women are a high‑risk population for severe dengue (OR = 3.03, 95% CI: [1.01, 9.08]).

2. Vaccine knowledge

**Fig 1:**
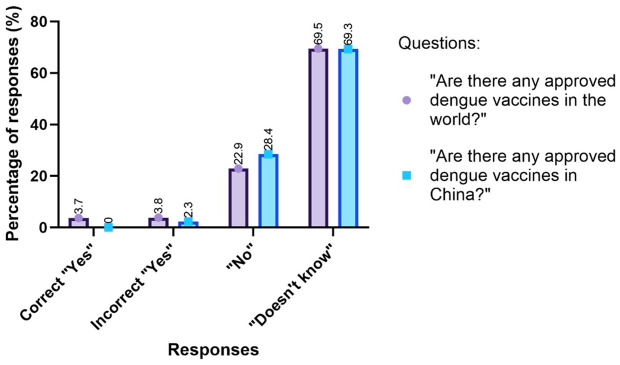
Responses to dengue vaccine approval questions.

79 respondents (7.5% of 1, 047) affirmed the existence of approved dengue vaccines globally (Fig 1). Among these, 39 participants (49.4%) correctly identified the approved dengue vaccines as Dengvaxia, Qdenga, or both, while 40 participants (50.6%) demonstrated incorrect knowledge (Fig 1). Regarding approved dengue vaccines in China, 297 respondents (28.4%) correctly indicated that none are approved. In contrast, 24 respondents (2.3%) incorrectly believed that a dengue vaccine was approved in China and provided incorrect vaccine names (Fig 1).

**Fig 2:**
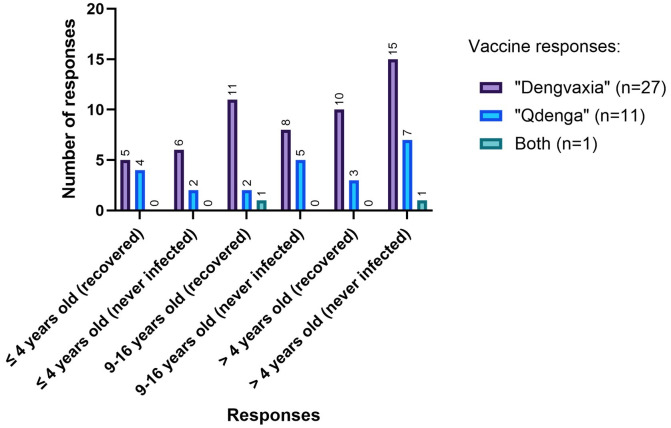
Accurate knowledge on vaccine-appropriate age population.

Among the 27 respondents who correctly named Dengvaxia as an approved vaccine, 11 selected the correct indication (“9 to 16 years old [recovered]”) as the appropriate age group for vaccination (Fig 2). However, only 4 of these 11 respondents selected this option exclusively (14.8% of the 27 respondents). Among the 11 respondents who correctly named Qdenga as an approved vaccine, 10 selected the correct indications (“older than 4 years old [recovered]” and “older than 4 years old [never infected]”) (Fig 2). However, only 2 respondents selected both options exclusively, reflecting that Qdenga can be administered to individuals aged >4 years regardless of dengue infection history (18.1% of the 11 respondents).

Only 1 respondent correctly named both vaccines but was not completely accurate on this parameter (Fig 2). However, the selection of “9 to 16 years old (recovered)” and “older than 4 years old (never infected)” reflected good knowledge of the vaccine-appropriate age population in general.

The majority of respondents demonstrated good understanding that dengue vaccination can reduce infection rates, re-infection rates, and hospitalization rates; the corresponding agreement proportions were 82.8%, 78.0%, and 83.3%, respectively (Table 2). However, more than half of the respondents (67.4%) incorrectly believed that dengue vaccination can prevent dengue virus infection (Table 2). Most respondents were uncertain about the number of doses needed for dengue vaccine administration and whether it offers lifelong immunity. Overall, 35.1% correctly understood that dengue vaccination does not confer lifelong immunity against the virus (Table 2).

**Table 2:** Knowledge on dengue vaccine (n = 1047).

	“Yes”	“No”	I don’t know
Dengue vaccine can reduce general infection rate	867 (82.8)	25 (2.4)	155 (14.8)
Dengue vaccine can reduce re-infection rate	817 (78.0)	50 (4.8)	180 (17.2)
Dengue vaccine can reduce hospitalization rate	872 (83.3)	30 (2.9)	145 (13.9)
Dengue vaccine can prevent dengue virus infection	706 (67.4)	138 (13.2)	203 (19.4)
Only 1 dose of dengue vaccine is needed for administration	324 (31.0)	204 (19.5)	519 (49.6)
Dengue vaccine offers life-long immunity	224 (21.4)	367 (35.1)	456 (43.6)

Among the 264 respondents who claimed to know about ADE, 46.2% were correct that not every dengue infection will induce an ADE response, 76.9% were correct that an ADE response results in severe dengue infection, and 66.7% were correct that an ADE response is induced when a recovered patient is re-infected by another serotype of dengue virus (Table 3). 45.8% of the 164 respondents were correct that recovered patients do not develop antibodies against all serotypes, and 56.4% were correct that antibodies in recovered patients aid the viral invasion of new serotypes of dengue virus into the host (Table 3). In general, the majority of the 264 respondents who claimed to know about ADE demonstrated accurate understanding of ADE associated with dengue virus.

**Table 3:** Knowledge on antibody-dependent enhancement (ADE).

Variable	Knows antibody-dependent enhancement (ADE)		
	Actually knows	Actually doesn’t know	Unsure
Every dengue infection will induce ADE response	122 (46.2)	98 (37.1)	44 (16.7)
ADE response results in severe dengue infection	203 (76.9)	25 (9.5)	36 (13.6)
ADE response is induced when a recovered patient is re-infected by another serotype of dengue virus	176 (66.7)	45 (17.1)	43 (16.3)
Recovered patients develop antibodies against all serotypes	121 (45.8)	110 (41.7)	33 (12.5)
Antibodies in recovered patients aid the viral invasion of new serotypes of dengue virus into host	149 (56.4)	70 (26.5)	45 (17.1)

3. Vaccine attitude

The majority of the respondents (87.6%) indicated that they would advocate for the vaccination of susceptible populations against dengue, should a vaccine be approved and accessible in China (Table 4). The inclination to recommend a dengue vaccine to the general population and those who have recovered from dengue was less pronounced, with rates of 52.2% and 45.2%, respectively (Table 4).

**Table 4:** Dengue vaccine attitudes (n = 1047).

Variables	Very Agree	Agree	No Opinion/I don’t know	Disagree	Very Disagree
Would you recommend the following to get vaccinated					
More susceptible populations	/	917 (87.6)	33 (3.2)	97 (9.3)	/
General population	/	547 (52.2)	258 (24.6)	242 (23.1)	/
Dengue-recovered patients	/	473 (45.2)	365 (34.9)	209 (20.0)	/
Attitude towards dengue safety and effectiveness					
Dengue vaccine is harmful to the body	120 (11.5)	88 (8.4)	369 (35.2)	348 (33.2)	122 (11.7)
Dengue vaccine can effectively reduce infection risk	324 (31.0)	460 (43.9)	224 (21.4)	31 (3.0)	8 (0.8)
Dengue vaccine has side effects	81 (7.7)	250 (23.9)	554 (52.9)	130 (12.4)	32 (3.01)
Dengue vaccine will trigger underlying diseases	73 (7.0)	94 (9.0)	492 (47.0)	312 (29.8)	76 (7.3)
Dengue vaccine has no long-term data to support its safety	84 (8.0)	235 (22.5)	549 (52.4)	141 (13.5)	38 (3.6)
Dengue vaccine has no long-term data to support effectiveness	79 (7.6)	238 (22.7)	551 (52.6)	144 (13.8)	35 (3.3)
The human body has its own immunity; dengue vaccination is not necessary	57 (5.4)	74 (7.1)	357 (34.1)	430 (41.1)	129 (12.3)
Dengue vaccination may serve scientific research purposes	91 (8.7)	206 (19.7)	537 (51.3)	169 (16.1)	44 (4.2)
Dengue vaccination may serve political purposes	53 (5.1)	66 (6.3)	423 (40.4)	375 (35.8)	130 (12.4)
If one doesn’t go out, one will not be infected with dengue virus; hence dengue vaccine is not needed	46 (4.4)	57 (5.4)	274 (26.2)	461 (44.0)	209 (20.0)
Dengue vaccination will affect fertility	49 (4.7)	48 (4.6)	450 (43.0)	367 (35.1)	133 (12.7)
Dengue vaccine may have negative impact on children’s development	50 (4.8)	57 (5.4)	486 (46.4)	331 (31.6)	123 (11.8)

Concerning the safety and efficacy of the dengue vaccine, a large percentage (74.9%) of the respondents expressed positivity that the vaccine could diminish the risk of infection. However, concerns regarding adverse effects were noted by 31.6% of participants (Table 4). Further issues included the potential aggravation of underlying diseases (16%), the absence of long-term safety data (30.5%), and a lack of information on long-term efficacy (30.3%) (Table 4). The impact of the vaccine on fertility was a concern for 9.3% of respondents, and a smaller yet considerable fraction of the surveyed population believed that natural immunity provides adequate protection against dengue (12.5%) or expressed the belief that the vaccine is intrinsically harmful (19.9%) (Table 4).

Additionally, some respondents held the view that dengue vaccination campaigns could be driven by motives unrelated to public health, such as research objectives (28.4%) or political agendas (11.4%) (Table 4). Misconceptions were observed, with 9.8% of participants suggesting that the absence of outdoor exposure negates the need for vaccination, and 10.2% expressing concerns about the impact of vaccines on child development (Table 4).

Further analysis was performed to identify the influence of perceived dengue knowledge, actual dengue knowledge, and actual vaccine knowledge on vaccine attitude. Respondents with perceived dengue knowledge were more likely to have a positive attitude towards the vaccine, with OR = 1.077. However, this association was statistically insignificant (95% CI: [0.30 – 3.90]).

**Fig 3:**
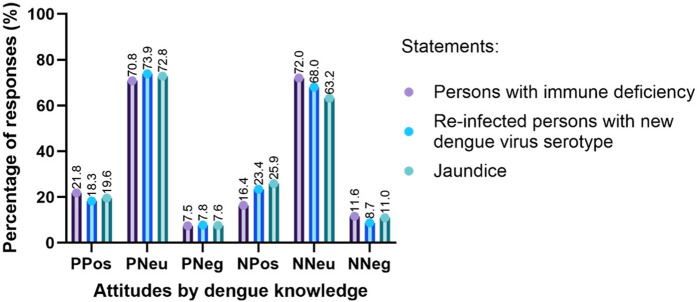
Dengue knowledge and vaccine attitude. PPos = Answered correctly and positive attitude; PNeu = Answered correctly and neutral attitude; PNeg = Answered correctly and negative attitude. NPos = Answered incorrectly and positive attitude; NNeu = Answered incorrectly and neutral attitude; NNeg = Answered incorrectly and negative attitude. Percentages are shown above bars.

The association between actual dengue knowledge and vaccine attitude was assessed for all knowledge domains, in which only two of the six domains yielded parameters with statistically significant results (Fig 3). These included knowledge of high at-risk population for severe dengue (severity) that covered immune-deficient individuals and re-infection of new virus serotype, and knowledge of infection symptoms that cover jaundice. Respondents were more likely to have a positive attitude towards dengue vaccine when they knew that persons with immune deficiency were high at-risk of severe dengue infection (OR = 1.785, 95% CI: [1.04, 3.05]) compared to those who were not knowledgeable on this. In contrast, respondents who knew that re‑infected individuals exposed to new dengue virus serotypes are at a higher risk of severe dengue infection were less likely to hold a positive attitude (OR = 0.558, 95% CI: [0.39, 0.81]) compared to those without this correct knowledge. Similarly, respondents that correctly knew jaundice to not be a common symptom were less likely to have a positive attitude (OR = 0.600, 95% CI: [0.37, 0.98]) compared to those without the correct knowledge.

Participant expresses positive attitude towards at least 8 out of 12 parameters. Participant expresses negative attitude towards at least 8 out of 12 parameters. Full table see S2 Table.

**Fig 4:**
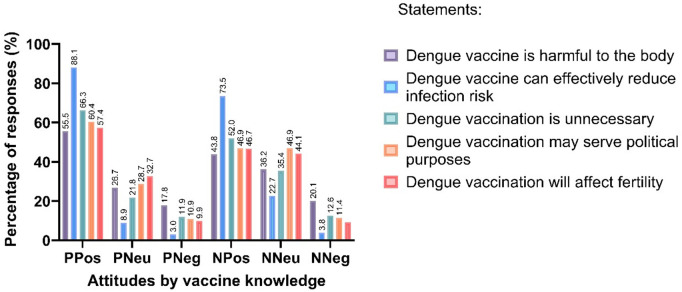
Vaccine knowledge and vaccine attitude. PPos = Knows vaccine and positive attitude; PNeu = Knows vaccine and neutral attitude; PNeg = Knows vaccine and negative attitude. NPos = Does not know vaccine and positive attitude; NNeu = Does not know vaccine and neutral attitude; NNeg = Does not know vaccine and negative attitude. Percentages are shown above bars.

Fig 4 presents the association between actual vaccine knowledge and vaccine attitude. Odds ratio assesses the likelihood that individuals with vaccine knowledge have a positive attitude compared to a neutral attitude, relative to those without vaccine knowledge. Out of the twelve parameters, five parameters yielded statistically significant results. Respondents with vaccine knowledge were more likely to have a positive attitude and disagree with the belief that the dengue vaccine is harmful to the body (OR = 1.713, 95% CI: [1.06, 2.77]), will affect fertility (OR = 1.658, 95% CI: [1.06, 2.60]), is unnecessary (OR = 2.074, 95% CI: [1.26, 3.42]), may serve political purposes (OR = 1.866, 95% CI: [1.18, 2.96]), and believe that the vaccine can effectively reduce infection risk (OR = 3.058, 95% CI: [1.52, 6.17]), compared to those without sufficient vaccine knowledge.

Know vaccine is defined as those that answered at least 4 out of the 6 questions correctly on basic dengue vaccine knowledge, and says that they know about antibody-dependent enhancement. Full table see S3 Table.

4. Anticipated practice

We assessed the distribution of vaccine acceptance in anticipated practices of vaccination, and the association between vaccine attitude and vaccine acceptance.

If dengue vaccines became available, 48% of respondents would accept vaccination themselves, while 46.8% would recommend it to family and friends. A quarter firmly rejected personal vaccination (25.6%) and recommendations to loved ones (23.4%), with the remainder being unsure (Table 5).

**Table 5:** Anticipated practice on dengue vaccine acceptance (N = 1047).

	Yes	I don’t know	No
Will take the dengue vaccine if available	503 (48.0)	276 (26.4)	268 (25.6)
Will recommend family and friends to take the dengue vaccine if available	490 (46.8)	312 (29.8)	245 (23.4)

Using chi-square tests, we found statistically significant differences (p < 0.001) across levels of vaccine acceptance and across vaccine attitude categories. The majority of respondents who accepted, refused, or had no opinion about vaccination had neutral attitudes toward the dengue vaccine (56.1%, 85.1%, and 84.3%, respectively) (S4 Table). Respondents who were willing to accept vaccines comprised the highest proportion across all attitude categories. Specifically, 73.9% of those with positive attitudes accepted vaccination, 38.0% of those with neutral attitudes accepted vaccination, and 69.8% of those with negative attitudes accepted vaccination (Table 6). The association between vaccine attitude and vaccine acceptance was supported by an OR of 3.946 (95% CI: [2.623, 5.937]).

**Table 6:** Dengue vaccine attitude by vaccine acceptance (N = 1047).

	Accept vaccine	No opinion	Refuse vaccine
Positive Attitude, n (%)	161 (73.9)	34 (15.6)	23 (10.6)
Neutral Attitude, n (%)	282 (38.0)	235 (31.6)	226 (30.4)
Negative Attitude, n (%)	60 (69.8)	7 (8.1)	19 (22.1)

## Discussion

This study assessed perceived and actual knowledge of dengue virus infection and vaccination among healthcare practitioners in China. The results revealed that, while a high proportion of participants perceived themselves as knowledgeable about dengue, their actual knowledge varied across domains. Participants who perceived themselves as knowledgeable about dengue were more likely to have accurate knowledge about the disease than their peers who did not consider themselves knowledgeable (mean OR = 1.38, median OR = 1.13).

Looking into the six knowledge domains: transmission mechanisms, mosquito vectors, exposure risk, severe dengue risk, symptoms, and complications yielded mixed results. The general trend demonstrates a positive association between perceived and actual knowledge across most domains, with mean OR > 1 (Table 1). The results also suggest that for the specific item on pregnant women as a high‑risk group for severe dengue, self‑perceived knowledge aligns with factual knowledge with statistical significance. However, for all other knowledge items, perceived knowledge was not significantly associated with actual knowledge, indicating that healthcare workers’ confidence in their dengue knowledge often does not correspond to accurate understanding. Besides statistical insignificance, the width of the 95% confidence intervals reflects the small sample sizes within the subgroups. Looking into the relationship between perceived and actual dengue knowledge across different domains, the most determinant domains of dengue awareness were knowledge of all other groups, except knowledge of the high-at-risk population for dengue exposure. To have the biggest possible impact, public health initiatives that disseminate information about the dengue virus may find it beneficial to focus on these areas or increase education for the populations that are likely to have dengue exposure. This would be beneficial for reducing exposure risks, for example, going outdoors without protection.

The investigation also revealed that respondents with perceived dengue knowledge were more likely to have a positive vaccine attitude. Although statistically insignificant, we cannot assume that there was no impact, as the results showed a consistent trend, suggesting some underlying pattern between the variables. The trend in the relationship between perceived dengue knowledge and vaccine attitude was potentially promising, affirmed by the positive relationship we yielded for the association between actual dengue knowledge and vaccine attitude (Fig 3, S2 Table). The general trend reflected a positive association, with only two domains yielding statistically significant parameters, which are the most determinant on vaccine attitude (Fig 3, S2 Table). Participants who knew that immunocompromised individuals are at high risk of severe dengue infection were more likely to hold a positive attitude toward the dengue vaccine. This finding aligns with our earlier observation that knowledge of at‑risk populations is a strong determinant of both accurate dengue knowledge and favorable vaccine attitudes. Hence, we have obtained a potentially promising direction to focus education campaigns and public awareness promotion projects.

From a public health perspective, the observation that 48% of respondents would accept a future dengue vaccine and 46.8% would recommend it to others indicates moderate but improvable acceptance. These figures highlight both an opportunity for vaccine promotion and the need to address remaining hesitancy. If Qdenga, with its reported efficacy of 59% against the dengue virus with R_0_ ~ 1.3, were to be used as the primary vaccine, the current level of acceptance might be considered to have achieved the herd immunity threshold [17–19]. However, to better prepare for seasonal peaks in dengue cases in endemic provinces, as well as considering the risk of severe dengue complications and the situation of ADE, it would be advantageous to strive for an even higher acceptance rate. A notable proportion – approximately one‑quarter of respondents – firmly rejected both personal vaccination (25.6%) and recommending the vaccine to family or friends (23.4%). This level of firm hesitancy highlights a significant barrier that would need to be addressed through targeted communication strategies and trust‑building measures prior to any future dengue vaccine rollout in China. Most respondents who accepted, refused, or had no opinion towards vaccines had a rather neutral attitude towards the dengue vaccine (S4 Table). Specifically, majority of respondents who would refuse the vaccine held a neutral attitude towards the vaccine (84.3%) (S4 Table). This suggests that vaccine refusal may not always be driven by strong negative attitudes towards the vaccine, but rather by other factors such as misconceptions or personal beliefs. This finding directs a path to focus education campaigns and vaccine promotion. Interestingly, respondents who were willing to accept vaccines occupied the highest proportion across all attitude categories. Acceptance of the dengue vaccine varied notably by attitude: 73.9% of those with a positive attitude accepted vaccination, compared to 38.0% of those with a neutral attitude (Table 6). Interestingly, 69.8% of participants with a negative attitude also accepted the vaccine – a finding that may reflect a disconnect between stated attitude and actual willingness, or possibly measurement issues that warrant further investigation. This phenomenon could be attributed to various factors, such as the perceived risk of dengue infection, the influence of healthcare providers, or the recognition of the benefits of vaccination despite personal reservations. The study findings also revealed that increasing knowledge about the dengue vaccine was associated with positive attitudes towards its safety and effectiveness, which could ultimately lead to greater vaccine acceptance (Fig 4). Therefore, public health authorities can focus on educating the public and high-risk groups about the risk factors for severe dengue infection and the common symptoms of the disease to promote positive attitudes towards vaccination, and address concerns and misconceptions about the vaccine.

Further studies can be conducted to better understand the complex relationship between knowledge, attitudes, and vaccine acceptance. These studies should aim to identify the key factors influencing vaccine hesitancy and develop evidence-based strategies to overcome barriers to vaccination. From the results in this study, we observed a trend in increased likelihood to have a positive attitude towards dengue vaccine when respondents were more knowledgeable in the virus itself, although the association was not statistically significant.

One limitation of the study lies in the unevenly distributed sample size between the two knowledge groups and hence has compromised the power of our findings. Our sample size of participants without perceived dengue knowledge was small (n = 15), and participants with knowledge of the dengue vaccine were not sufficient (n = 101) when compared to participants without dengue vaccine knowledge (n = 946). This may have affected the statistical power and generalizability of the findings. Future studies should aim to recruit a more balanced and larger sample, which could also potentially resolve the challenge of a wide 95% confidence interval range that we obtained in this study. In terms of validity, the study has higher internal validity but limited external validity because the study was conducted in specific provinces in China and specifically designed for healthcare practitioners, and the findings may not be generalizable to other settings or populations. Future investigations should include a broader range of participants from the dengue endemic regions to assess the consistency of knowledge and vaccine acceptance across different populations. In addition, the present study did not investigate whether demographic or professional characteristics (such as age, work title, or department) predict healthcare workers’ willingness to recommend a future dengue vaccine, although we did examine the role of vaccine knowledge. Future research should therefore explore how these population characteristics influence recommendation intentions to help tailor communication strategies and address specific barriers within different subgroups.

By increasing awareness and knowledge on determinant knowledge domains, addressing misconceptions and concerns through targeted interventions and effective communication, public health authorities can work towards increasing dengue vaccine uptake and ultimately reducing the burden of dengue disease. We believe that the introduction and research for the dengue virus vaccine would bring beneficial impacts for the Chinese population. The findings of this study offer a data‑driven foundation for policy decisions regarding dengue vaccine development and deployment in China, as well as practical guidance for crafting education campaigns that directly target the knowledge domains most strongly associated with vaccine acceptance.

## Supporting information

S1 Table Baseline Characteristics between different knowledge status groups on dengue virus. The table compares the demographic and professional characteristics of healthcare workers who self‑reported knowing dengue (“Yes,” n = 1,032) versus those who reported no knowledge (“No,” n = 15). Continuous variables (age, years of working) are presented as mean ± standard deviation (SD) and were compared using independent t‑tests. Categorical variables (work department, work title, education level) are presented as counts (n) and percentages (%) and were compared using chi‑square tests. For each variable, the degrees of freedom (df) and p‑values are provided. Statistical significance was set at p < 0.05.
(DOCX)

S2 Table Actual dengue knowledge and dengue vaccine attitude. This table presents the relationship between participants’ factual knowledge of dengue (answered correctly vs. incorrectly) across six knowledge domains and their attitude toward dengue vaccination. The six domains are: (1) dengue transmission, (2) mosquito vectors, (3) high‑risk populations for dengue exposure, (4) high‑risk populations for severe dengue, (5) infection symptoms, and (6) possible infection complications. Vaccine attitude was categorized as positive (participant expressed favorable views on at least 8 out of 12 attitude parameters), negative (unfavorable views on at least 8 out of 12 parameters), or neutral (all other responses). Counts (n) and column percentages (%) are shown for each combination of knowledge (correct/incorrect) and attitude category. Odds ratios (OR) with 95% confidence intervals (CI) estimated using univariable ordinal logistic regression with maximum likelihood to assess the association between correct knowledge (vs. incorrect) and positive attitude (vs. negative) based on neutral responses.
(DOCX)

S3 Table Actual vaccine knowledge and dengue vaccine attitude. This table presents the relationship between participants’ factual knowledge of the dengue vaccine and their attitude toward dengue vaccination. The knowledge statements cover vaccine effectiveness, side effects, necessity, and general vaccine facts. “Know vaccine” was defined as: (1) answering at least 4 out of 6 questions correctly on basic dengue vaccine knowledge, and (2) stating that they know about antibody‑dependent enhancement (ADE). Participants who did not meet both criteria were classified as “not know vaccine”. Vaccine attitude was categorized as positive, neutral, or negative using the same 12‑parameter definition described in S2 Table. Counts (n) and column percentages (%) are shown for each knowledge‑attitude combination. Ordinal logistic regression (generalized logit) using maximum likelihood estimation (MLE) was used. For each statement, the model estimated the effect of vaccine knowledge (knows vs. not know) on the odds of having a positive attitude versus a neutral attitude, presented as OR (95% CI).
(DOCX)

S4 Table Dengue vaccine attitude by vaccine acceptance. This table cross‑tabulates participants’ overall dengue vaccine attitude (positive, neutral, or negative, as defined in S2 Table) with their stated willingness to accept a future dengue vaccine (accept, no opinion, refuse). Counts (n) and row percentages (%) are presented for each attitude‑acceptance combination. The table allows visual inspection of the association between attitude and acceptance; no statistical test is reported here.
(DOCX)
