# Supplementary material for: Evaluating knowledge and attitudes towards dengue fever and its vaccination in China: A questionnaire-based study in healthcare practitioners
Source: PLoS Negl Trop Dis. 2026 Aug 5;20(8):e0013530. doi: 10.1371/journal.pntd.0013530 (PMC13472495; doi:10.1371/journal.pntd.0013530)
Supplement: Supplementary file 1 [file pntd.0013530.s001.docx]

**S1 Table. Baseline Characteristics between different knowledge status groups on dengue virus**

| Variables | Dengue knowledge | | p (DF) ^a^ |
| --- | --- | --- | --- |
|  | **Yes (n=1032)** | **No (n=15)** |  |
| Age (years), mean ± SD | 38.4 ± 9.0 | 42.8 ± 10.1 | 0.065 (DF=1045) |
| Years of working, mean ± SD | 3.1 ± 1.5 | 3.7 ± 1.4 | 0.119 (DF=1045) |
| Work Department, n (%) |  |  | 0.066 (DF=3) |
| Centers for Disease Control and Prevention | 297 (28.8) | 9 (60.0) |  |
| Community Health Service/ Health Center | 686 (66.5) | 6 (40.0) |  |
| Secondary Hospital | 31 (3.0) | 0 (0.0) |  |
| Tertiary Hospital | 18 (1.7) | 0 (0.0) |  |
| Work Title, n (%) |  |  | 0.431 (DF=4) |
| No title | 128 (12.4) | 3 (20.0) |  |
| Junior | 336 (32.6) | 2 (13.3) |  |
| Intermediate | 416 (40.3) | 6 (40.0) |  |
| Vice-senior | 118 (11.4) | 3 (20.0) |  |
| Senior | 34 (3.3) | 1 (6.7) |  |
| Education Level, n (%) |  |  | 0.012 (DF=4) |
| Middle school and below | 4 (0.4) | 1 (6.7) |  |
| High school, vocational high school, technical secondary school, technical school | 36 (3.5) | 1 (6.7) |  |
| College | 168 (16.3) | 2 (13.3) |  |
| Undergraduate | 767 (74.3) | 10 (66.7) |  |
| Postgraduate | 57 (5.5) | 1 (6.7) |  |

**^a^** p-value: chi-square test is used for categorical variables; t-test is used for continuous variables.
