# Supplementary material for: Evaluating knowledge and attitudes towards dengue fever and its vaccination in China: A questionnaire-based study in healthcare practitioners
Source: PLoS Negl Trop Dis. 2026 Aug 5;20(8):e0013530. doi: 10.1371/journal.pntd.0013530 (PMC13472495; doi:10.1371/journal.pntd.0013530)
Supplement: Supplementary file 2 [file pntd.0013530.s002.docx]

**S2 Table. Actual dengue knowledge and dengue vaccine attitude**

| Statements | Answered correctly | | | Answered incorrectly | | | Odds ratio  (95% CI) |
| --- | --- | --- | --- | --- | --- | --- | --- |
|  | **Positive attitude ^a^**  **N (%)** | **Neutral attitude**  **N (%)** | **Negative attitude ^b^**  **N (%)** | **Positive attitude ^a^**  **N (%)** | **Neutral attitude**  **N (%)** | **Negative attitude ^b^**  **N (%)** |  |
| Knowledge of dengue transmission | | | | | | | |
| Sexual transmission | 196 (20.92) | 667 (71.2) | 74 (7.9) | 22 (20.0) | 76 (69.1) | 12 (10.9) | 0.977  [0.49, 1.97] |
| Mosquito bite^c^ | 218 (21.0) | 737 (70.9) | 84 (8.1) | 0 (0.0) | 6 (75.0) | 2 (25.0) | 3.895  [0.22, 70.36] |
| Direct contact | 181 (21.3) | 611 (71.8) | 59 (6.9) | 37 (18.9) | 132 (67.4) | 27 (13.8) | 0.926  [0.54, 1.60] |
| Indirect contact | 187 (21.0) | 629 (70.8) | 73 (8.2) | 31 (19.6) | 114 (72.2) | 13 (8.2) | 1.421  [0.77, 2.63] |
| Saliva transmission | 189 (20.9) | 642 (70.9) | 74 (8.2) | 29 (20.4) | 101 (71.1) | 12 (8.5) | 1.031  [0.56, 1.89] |
| Blood transmission | 137 (21.4) | 450 (70.4) | 52 (8.1) | 81 (19.9) | 293 (71.8) | 34 (8.3) | 1.270  [0.87, 1.85] |
| Vertical transmission | 28 (26.7) | 71 (67.7) | 6 (5.7) | 190 (20.2) | 672 (71.3) | 80 (8.5) | 1.637  [0.88, 3.03] |
| Human-mosquito | 66 (20.2) | 229 (70.0) | 32 (9.8) | 152 (21.1) | 514 (71.4) | 54 (7.5) | 0.972  [0.68, 1.39] |
| Knowledge of mosquito vectors | | | | | | | |
| Anopheles sinensis | 181 (21.7) | 595 (71.3) | 58 (7.0) | 37 (18.0) | 143 (69.4) | 26 (12.6) | 1.270  [0.74, 2.19] |
| Anopheles anthropophila | 204 (21.1) | 687 (71.2) | 74 (7.7) | 14 (18.7) | 51 (68.0) | 10 (13.3) | 1.130  [0.40, 3.16] |
| Anopheles anopheles | 203 (20.9) | 695 (71.4) | 75 (7.7) | 15 (22.4) | 43 (64.2) | 9 (13.4) | 0.427  [0.15, 1.20] |
| Culex pipiens | 199 (21.0) | 675 (71.4) | 72 (7.6) | 19 (20.2) | 63 (67.0) | 12 (12.8) | 0.901  [0.43, 1.98] |
| Culex tritaenorhynchus | 189 (20.8) | 657 (72.1) | 65 (7.1) | 29 (22.5) | 811 (62.8) | 19 (14.7) | 0.738  [0.41, 1.33] |
| Aedes albopictus | 200 (21.6) | 660 (71.1) | 68 (7.3) | 18 (16.1) | 78 (69.6) | 16 (14.3) | 1.620  [0.86, 3.07] |
| Aedes aegypti | 182 (22.0) | 588 (71.0) | 58 (7.0) | 36 (17.0) | 150 (70.8) | 26 (12.3) | 1.244  [0.79, 1.97] |
| Chironomid | 210 (21.1) | 707 (71.1) | 77 (7.8) | 8 (17.4) | 31 (67.4) | 7 (15.2) | 2.728  [0.79, 9.46] |
| Knowledge of high at-risk population for dengue (exposure) | | | | | | | |
| Elderly | 90 (21.7) | 299 (72.2) | 25 (6.0) | 128 (20.2) | 444 (70.1) | 61 (9.6) | 0.912  [0.50, 1.65] |
| Infants and young children | 97 (22.4) | 307 (70.9) | 29 (6.7) | 121 (19.7) | 436 (71.0) | 57 (9.3) | 1.470  [0.83, 2.60] |
| Pregnant women | 119 (20.5) | 414 (71.3) | 48 (8.3) | 99 (21.2) | 329 (70.6) | 38 (8.2) | 0.740  [0.41, 1.32] |
| Patients with underlying diseases | 94 (21.3) | 322 (72.9) | 26 (5.9) | 124 (20.5) | 421 (69.6) | 60 (9.9) | 0.949  [0.55, 1.64] |
| Persons with immune deficiency | 81 (22.3) | 257 (70.8) | 25 (6.9) | 137 (20.0) | 486 (71.1) | 61 (8.9) | 1.494  [0.91, 2.45] |
| Re-infected persons with new dengue virus serotype | 115 (20.3) | 413 (72.7) | 40 (7.0) | 103 (21.5) | 330 (68.9) | 46 (9.6) | 0.696  [0.47, 1.03] |
| Smoker | 182 (20.5) | 639 (72.0) | 66 (7.4) | 36 (22.5) | 104 (65.0) | 20 (12.5) | 0.736  [0.39, 1.40] |
| Persons with malnutrition | 144 (20.9) | 491 (71.2) | 55 (8.0) | 74 (20.7) | 252 (70.6) | 31 (8.7) | 1.146  [0.70, 1.88] |
| Outdoor workers | 167 (22.1) | 548 (72.4) | 42 (5.6) | 51 (17.6) | 195 (67.2) | 44 (15.2) | 1.237  [0.791, 1.93] |
| Returning travelers | 120 (21.1) | 419 (73.5) | 31 (5.4) | 98 (20.6) | 324 (67.9) | 55 (11.5) | 0.679  [0.45, 1.02] |
| Knowledge of high at-risk population for severe dengue (severity) | | | | | | | |
| Elderly | 171 (21.0) | 578 (70.9) | 66 (8.1) | 47 (20.3) | 165 (71.1) | 20 (8.6) | 1.212  [0.70, 2.09] |
| Infants and young children | 141 (20.1) | 502 (71.7) | 57 (8.1) | 77 (22.2) | 241 (69.5) | 29 (8.4) | 0.658  [0.39, 1.12] |
| Pregnant women | 118 (20.9) | 400 (70.9) | 46 (8.2) | 100 (20.7) | 343 (71.0) | 40 (8.3) | 1.057  [0.65, 1.73] |
| Patients with underlying diseases | 175 (20.6) | 607 (71.6) | 66 (7.8) | 43 (21.6) | 136 (68.3) | 20 (10.1) | 0.673  [0.40, 1.13] |
| Persons with immune deficiency | 187 (21.8) | 607 (70.8) | 64 (7.5) | 31 (16.4) | 136 (72.0) | 22 (11.6) | 1.785  [1.04, 3.05] |
| Re-infected persons with new dengue virus serotype | 97 (18.3) | 391 (73.9) | 41 (7.8) | 121 (23.4) | 352 (68.0) | 45 (8.7) | 0.558  [0.39, 0.81] |
| Smoker | 192 (21.0) | 653 (71.3) | 71 (7.8) | 26 (19.9) | 90 (68.7) | 15 (11.5) | 1.066  [0.55, 2.06] |
| Persons with malnutrition | 94 (22.2) | 300 (70.8) | 30 (7.1) | 124 (19.9) | 443 (71.1) | 56 (9.0) | 1.198  [0.81, 1.78] |
| Outdoor workers | 160 (21.3) | 536 (71.3) | 56 (7.5) | 58 (19.7) | 207 (70.2) | 30 (10.2) | 1.009  [0.61, 1.68] |
| Returning travelers | 178 (21.2) | 595 (70.9) | 66 (7.9) | 40 (19.2) | 148 (71.2) | 20 (9.6) | 1.012  [0.55, 1.87] |
| Knowledge of infection symptoms | | | | | | | |
| Fever | 217 (21.0) | 735 (71.2) | 81 (7.8) | 1 (7.1) | 8 (57.1) | 5 (35.7) | 3.255  [0.36, 29.21] |
| Nausea/Vomiting | 114 (20.6) | 382 (69.1) | 57 (10.3) | 104 (21.1) | 361 (73.1) | 29 (5.9) | 0.904  [0.61, 1.35] |
| Pain behind eyes | 157 (22.0) | 507 (70.9) | 51 (7.1) | 61 (18.4) | 236 (71.1) | 35 (10.5) | 1.209  [0.82, 1.78] |
| Runny nose | 159 (20.8) | 552 (72.2) | 54 (7.1) | 59 (21.0) | 191 (67.7) | 32 (11.4) | 1.109  [0.70, 1.77] |
| Abdominal pain | 100 (22.9) | 297 (68.1) | 39 (8.9) | 118 (19.3) | 446 (73.0) | 47 (7.7) | 1.337  [0.88, 2.04] |
| Jaundice | 166 (19.6) | 616 (72.8) | 64 (7.6) | 52 (25.9) | 1217 (63.2) | 22 (11.0) | 0.600  [0.37, 0.98] |
| Rash | 165 (21.2) | 559 (71.9) | 53 (6.8) | 53 (19.6) | 184 (68.2) | 33 (12.2) | 0.804  [0.53, 1.22] |
| Bleeding gums | 112 (23.0) | 343 (70.3) | 33 (6.8) | 106 (19.0) | 400 (71.6) | 53 (9.5) | 1.194  [0.83, 1.73] |
| Bone and joint pain | 162 (21.5) | 546 (72.4) | 46 (6.1) | 56 (19.1) | 197 (67.2) | 40 (13.7) | 0.840  [0.56, 1.27] |
| Knowledge of possible infection complications | | | | | | | |
| Acute myocarditis/ Acute heart failure | 206 (21.6) | 666 (70.0) | 80 (8.4) | 122 (12.6) | 77 (81.1) | 6 (6.3) | 2.290  [1.17, 4.49] |
| Pulmonary failure | 104 (20.2) | 360 (69.8) | 52 (10.1) | 114 (21.5) | 383 (72.1) | 34 (6.4) | 0.888  [0.60, 1.32] |
| Liver failure | 147 (20.3) | 516 (71.2) | 62 (8.6) | 71 (22.1) | 227 (70.5) | 24 (7.5) | 0.816  [0.53, 1.25] |
| Acute renal failure | 168 (20.7) | 581 (71.6) | 63 (7.8) | 50 (21.3) | 162 (68.9) | 23 (9.8) | 0.838  [0.54, 1.30] |
| Encephalopathy/ Encephalitis | 150 (22.4) | 467 (69.6) | 54 (8.1) | 68 (18.1) | 276 (73.4) | 32 (8.5) | 1.265  [0.86, 1.86] |
| Acute gastroenteritis | 63 (20.1) | 219 (69.8) | 32 (10.2) | 155 (21.2) | 524 (71.5) | 54 (7.4) | 0.866  [0.56, 1.34] |

^a^ Participant expresses positive attitude towards at least 8 out of 12 parameters. ^b^ Participant expresses negative attitude towards at least 8 out of 12 parameters. ^c^Haldane-Ascombre correction is applied by adding 0.5 to variables with cells containing 0 counts for odds ratio calculation. This variable is not included in interpretation due to a wide confidence interval range.
