# Supplementary material for: Evaluating knowledge and attitudes towards dengue fever and its vaccination in China: A questionnaire-based study in healthcare practitioners
Source: PLoS Negl Trop Dis. 2026 Aug 5;20(8):e0013530. doi: 10.1371/journal.pntd.0013530 (PMC13472495; doi:10.1371/journal.pntd.0013530)
Supplement: Supplementary file 3 [file pntd.0013530.s003.docx]

**S3 Table. Vaccine knowledge and dengue vaccine attitude**

| Statements | Knows vaccine ^a^ | | | Not know vaccine | | | Odds Ratio^b^  (95% CI) |
| --- | --- | --- | --- | --- | --- | --- | --- |
|  | **Positive attitude** | **Neutral attitude** | **Negative attitude** | **Positive attitude** | **Neutral attitude** | **Negative attitude** |  |
| Dengue vaccine is harmful to the body | 56 (55.5) | 27 (26.7) | 18 (17.8) | 414 (43.8) | 342 (36.2) | 190 (20.1) | 1.713  [1.06, 2.77] |
| Dengue vaccine can effectively reduce infection risk | 89 (88.1) | 9 (8.9) | 3 (3.0) | 695 (73.5) | 215 (22.7) | 36 (3.8) | 3.058  [1.52, 6.17] |
| Dengue vaccine has side effects | 18 (17.8) | 43 (42.6) | 40 (39.6) | 144 (15.2) | 511 (54.0) | 291 (30.8) | 1.486  [0.83, .66] |
| Dengue vaccine will trigger underlying diseases | 41 (41.6) | 43 (42.6) | 17 (16.8) | 347 (36.7) | 449 (47.5) | 150 (15.9) | 1.234  [0.79, 1.94] |
| Dengue vaccine has no long-term data to support its safety | 16 (15.8) | 54 (53.5) | 31 (30.7) | 163 (17.2) | 495 (52.3) | 288 (30.4) | 0.900  [0.50, 1.62] |
| Dengue vaccine has no long-term data to support effectiveness | 17 (16.8) | 50 (49.5) | 34 (33.7) | 162 (17.1) | 501 (53.0) | 283 (29.9) | 1.051  [0.59, 1.87] |
| The human body has its own immunity; dengue vaccination is not necessary | 67 (66.3) | 22 (21.8) | 12 (11.9) | 492 (52.0) | 335 (35.4) | 119 (12.6) | 2.074  [1.26, 3.42] |
| Dengue vaccination may serve scientific research purposes | 26 (25.7) | 43 (42.6) | 32 (31.7) | 187 (19.8) | 494 (52.2) | 265 (28.0) | 1.597  [0.95, 2.67] |
| Dengue vaccination may serve political purposes | 61 (60.4) | 29 (28.7) | 11 (10.9) | 444 (46.9) | 394 (46.9) | 108 (11.4) | 1.866  [1.18, 2.96] |
| If one doesn’t go out, one will not be infected with dengue virus; hence dengue vaccine is not needed | 72 (71.3) | 20 (19.8) | 9 (8.9) | 598 (63.2) | 254 (26.9) | 94 (9.9) | 1.529  [0.91, 2.56] |
| Dengue vaccination will affect fertility | 58 (57.4) | 33 (32.7) | 10 (9.9) | 442 (46.7) | 417 (44.1) | 87 (9.2) | 1.658  [1.06, 2.60] |
| Dengue vaccine may have negative impact on children’s development | 54 (53.5) | 40 (39.6) | 7 (6.9) | 400 (42.3) | 446 (47.2) | 100 (10.6) | 1.505  [0.98, 2.32] |

^a^ Know vaccine is defined as those who answered at least 4 out of the 6 questions on basic dengue vaccine knowledge and said that they know about antibody-dependent enhancement. ^b^ Odds ratio is assessing the likelihood that individuals with vaccine knowledge have a positive attitude compared to a negative attitude, relative to those without vaccine knowledge
