# Supplementary material for: Evaluating knowledge and attitudes towards dengue fever and its vaccination in China: A questionnaire-based study in healthcare practitioners
Source: PLoS Negl Trop Dis. 2026 Aug 5;20(8):e0013530. doi: 10.1371/journal.pntd.0013530 (PMC13472495; doi:10.1371/journal.pntd.0013530)
Supplement: Supplementary file 4 [file pntd.0013530.s004.docx]

**S4 Table. Dengue vaccine attitude by vaccine acceptance**

|  | Accept vaccine  N (%) | No opinion  N (%) | Refuse vaccine  N (%) |
| --- | --- | --- | --- |
| Positive Attitude | 161 (32.0) | 34 (12.3) | 23 (8.6) |
| Neutral Attitude | 282 (56.1) | 235 (85.1) | 226 (84.3) |
| Negative Attitude | 60 (11.9) | 7 (2.5) | 19 (7.1) |
